# Organ‐on‐a‐Chip Technology and Global Multi‐Omics: Current Applications and Future Directions

**DOI:** 10.1002/mco2.70603

**Published:** 2026-01-31

**Authors:** Xuxia Cao, Congmin Xia, Caifeng Li, Shiwen Deng, Junxian Cao, Hongjun Yang, Shaoping Wang, Peng Chen

**Affiliations:** ^1^ Experimental Research Center China Academy of Chinese Medical Sciences Beijing China; ^2^ BinZhou Medical University Yantai City China; ^3^ Guang 'anmen Hospital China Academy of Chinese Medical Sciences Beijing China; ^4^ Institute of Traditional Chinese Medicine Health Industry China Academy of Chinese Medical Sciences Nanchang China; ^5^ Analysis of Complex Effects of Proprietary Chinese Medicine Hunan Provincial Key Laboratory Yongzhou China

**Keywords:** disease mechanism, drug discovery, multi‐omics technology, organ‐on‐a‐chip

## Abstract

Biomedical research models are undergoing continuous evolution, while conventional models (two‐dimensional/ three‐dimensional cultures and animal studies) face limitations in physiological relevance and ethical constraints. Against this backdrop, the integration of organ‐on‐a‐chip (OoC) technology with multi‐omics methodologies is driving a profound paradigm shift in the field. OoC platforms utilize microfluidic technology to construct biomimetic three‐dimensional microenvironments capable of highly simulating human physiological and pathological states, while multi‐omics technologies (e.g., proteomics, transcriptomics, and metabolomics) provide systematic molecular profiling capabilities. The integration of these two approaches enables multi‐scale mechanistic analysis from molecular networks to the tissue level, significantly enhancing their potential in drug development and personalized medicine strategies. This article systematically reviews the research progress and existing challenges in this interdisciplinary field, with a focus on: (1) The developmental trajectory of OoC platforms from two‐dimensional to biomimetic three‐dimensional systems; (2) mechanistic insights revealed by the integration of multi‐omics and OoC technology in modeling disease processes; and (3) key issues in the standardization and clinical translation of OoC technology. Finally, the paper proposes a development roadmap for constructing next‐generation disease models, aiming to provide a theoretical framework and strategic guidance for the establishment of standardized systems and clinical translation pathways in this field.

## Graphical abstract

**Comparative analysis of multiple cell culture models**. The 3D microfluidic organ‐on‐a‐chip, which is superior to the existing models in multiple aspects, can be more conducive to new studies and discoveries related to human beings. Created with BioRender (www.biorender.com).

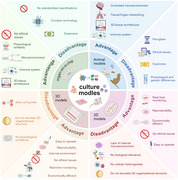

## 1 Introduction

The evolution of biomedical research models has primarily encompassed key stages including two‐dimensional/three‐dimensional (2D/3D) cell culture models, and animal models [1, 2]. However, they all have obvious limitations. The 2D cell culture model fails to accurately mimic the complex cellular niche and physiological processes in vivo owing to the lack of key factors, including extracellular matrix (ECM), physicochemical and biological clues, as well as multicellular interactions and intracellular signaling pathways [3, 4]. Subsequently developed 3D cell culture models have made progress in simulating the three‐dimensional structure and function of cells. However, they still struggle to accurately replicate the complex geometric architecture and dynamic mechanical properties of specific organs, such as the lungs. In addition, these models often face challenges such as necrotic cell death in the core region and poor controllability of cell–cell interactions, which limit their predictive accuracy [5, 6]. While animal models can provide a holistic physiological context, they are constrained by inherent limitations, including long experimental cycles, high costs, and ethical controversies. Most critically, due to genetic and physiological differences between species, these models often fail to accurately recapitulate the pathological processes of human diseases and specific drug responses. This discrepancy directly contributes to the high failure rate observed in the transition from preclinical studies to clinical trials [7, 8, 9]. Thus, new alternative models and technologies are urgently needed to address these challenges.

Given the numerous challenges faced by traditional research models, OoC technology has emerged, bringing new hope to the study of diseases. OoCs are a disruptive technology that integrates multidisciplinary knowledge including bioengineering, microfluidics, materials science, and cell biology [10, 11]. The core concept involves creating 3D cell culture microenvironments within microfluidic chips and precisely controlling biomechanical stimuli, such as fluid shear stress and mechanical stretching, with the goal of closely mimicking the key structures, microenvironment, and physiological functions of human organs [12, 13, 14].Compared with traditional 2D or 3D culture systems, the core advantage of OoCs lies in its ability to achieve superior physiological relevance. It can not only recapitulate tissue‐level complex structures (such as the alveolar–capillary barrier) but also simulate critical physiological processes (e.g., blood flow, respiratory movements) through dynamic perfusion. Thereby, it overcomes the fundamental limitations of static culture systems, which fail to replicate complex cell–cell interactions and the dynamic microenvironment in vivo [15, 16]. Moreover, compared to animal models, OoC platforms utilize human‐derived cells, which directly circumvents the distortion of physiological and pathological responses caused by species differences. Thereby, it provides a more precise platform for investigating human‐specific biological processes [17]. These advantages endow it with significant potential in modeling human physiology and pathology. Taking the lung‐on‐a‐chip as an example, it can accurately simulate key pulmonary functions—including gas exchange, immune responses, and specific pathological states—playing an indispensable role in elucidating disease mechanisms and advancing drug screening [18]. Moreover, its construction and application framework demonstrate broad applicability across various organ systems. For instance, the “cartilage‐on‐a‐chip” developed by Zhao et al. for osteoarthritis research, which successfully established a disease model by optimizing GelMA hydrogel scaffolds, microfluidic perfusion, and inflammatory cytokine induction, offers a replicable paradigm for other OoCs [19]. Furthermore, the integration of OoCs with technologies like patient‐derived organoids is now advancing the development of personalized medicine [20]. Studies by Zhang et al. [21] on cholangiocarcinoma using patient‐derived organoids have shown that these highly faithful models display drug responses correlating closely with clinical outcomes, highlighting the promise of chip‐based systems for personalized therapy prediction and guidance. Significant advances have now been made in OoC technology, evidenced by the successful development of various OoCs (e.g., vascular, lung, kidney, brain, heart, intestinal, and skin) for simulating organ functions and modeling diseases [22, 23, 24, 25, 26, 27, 28, 29, 30], marking the technology's growing maturity.

The advent of global multi‐omics technologies marks the official entry of life sciences research into a new era of systems biology [31]. This technological framework encompasses genomics, transcriptomics, proteomics, metabolomics, epigenomics, and other dimensions, enabling systematic decoding of biological processes through comprehensive analysis of biomolecular profiles [32, 33, 34]. Genomics establishes the genetic background, while transcriptomics captures dynamic gene expression. Proteomics portrays the functional execution state, with metabolomics reflecting end‐point physiological responses and epigenomics elucidating the regulatory mechanisms governing gene expression [35]. The core advantage of multi‐omics lies in its integrative capacity—by correlating molecular information across different levels, it constructs a complete regulatory network from genotype to phenotype, thereby achieving a systematic and comprehensive decoding of biological processes [36, 37]. Understanding, at the network level, how molecular synergies regulate complex biological processes such as health maintenance, disease initiation, and drug response [38, 39]. This holistic perspective not only deepens our understanding of life's complexity but also establishes a solid foundation for elucidating disease mechanisms and developing precision diagnostics and therapeutic strategies.

The integration of OoC technology with multi‐omics approaches has enabled a paradigm shift in scientific research—from static “structural simulation” to dynamic “functional analysis”—providing unprecedented depth and breadth for elucidating disease mechanisms and evaluating drug responses [40]. Through microengineering‐based designs, OoCs replicate the physiological microenvironment, 3D architecture, and biomechanical characteristics of human organs, thereby reconstructing key physiological and pathological phenotypes and offering a highly simulated biological context for molecular mechanistic studies [41, 42, 43]. Multi‐omics technologies serve as a “molecular microscope” within these biomimetic settings. By systematically monitoring genomic, transcriptomic, proteomic, and metabolomic layers, they dynamically capture temporal and spatial changes in gene expression, signaling pathways, and metabolic networks, thereby deciphering the molecular drivers underlying phenotypic observations. In cancer research, OoC models constructed from patient‐derived cells not only simulate the structural features of the tumor microenvironment but also, when coupled with multi‐omics analysis, identify key gene mutations, signaling pathway activation, and metabolic reprogramming events before and after drug treatment [44]. This enables in‐depth interpretation of individualized drug response mechanisms [45].In drug toxicity assessment, lung chips can reproduce drug absorption and metabolic processes in pulmonary tissue, while transcriptomic and proteomic analyses systematically reveal oxidative stress, inflammatory signaling, and apoptotic networks involved in toxic responses, providing molecular evidence for early safety evaluation. For example, the ALI‐OLMP platform combined RNA‐Seq and iTRAQ technologies to dissect the toxicity mechanisms of PM2.5 [46]. Furthermore, by modeling lung cancer brain metastasis in multi‐organ chips, researchers have utilized multi‐omics approaches to identify key molecular signatures regulating drug resistance in resistant cell subpopulations (e.g., PC9‐Br), achieving a systematic interpretation of metastatic drug resistance mechanisms [47]. With the ongoing integration of organoids, 3D bioprinting, and other advanced technologies with OoC platforms [48, 49, 50], this convergent system not only demonstrates high biomimicry and scalability but also, powered by multi‐level data integration, drives biomedical research beyond localized phenotypic observation toward a mechanism‐driven systemic understanding—thereby injecting new momentum into precision medicine and drug development [51].

This review will focus on the integration of OoCs and multi‐omics technologies, systematically elaborating key technical methodologies, detailing representative applications in disease modeling and drug development, and discussing future challenges and development trends. It aims to provide a clear research pathway and perspective for advancing this cutting‐edge interdisciplinary field.

## 2 The Evolution and Utilization of OoC Technology

Conventional in vitro cell cultures (2D/3D) and animal models struggle to meet the demands of biomedical research due to limitations such as inadequate physiological relevance and interspecies differences. Against this backdrop, OoC technology has emerged as a transformative solution. This section systematically outlines the developmental trajectory of OoC technology—starting with an analysis of the limitations of traditional in vitro culture models, elaborating on the background and core design principles underlying the advent of OoCs, detailing their specific applications in the research of diseases such as pneumonia and lung cancer, and further extending to their practical value in drug development and clinical translation. It comprehensively demonstrates how this technology overcomes the bottlenecks of traditional models and has become a pivotal tool bridging basic research and clinical application.

### 2.1 Conventional in Vitro Cell Culture Approach

The 2D cell culture technology that emerged in the 1960s has held [52] and can be used for drug screening and basic research in cell biology [53, 54, 55]. But as research deepens, its limitations become apparent, since usually only one type of cell can be cultured, making it difficult to construct a multi‐cell interaction microenvironment in the lungs, reducing cell activity and experimental accuracy [56, 57, 58]. The difference between 2D culture results and 3D culture and in vivo environment results is significant, and these results cannot accurately reflect the growth characteristics and mechanisms of lung cancer in its true physiological state [59].

To overcome the shortcomings of 2D cell culture, 3D cell culture models have emerged. It constructs models that are more physiologically relevant to human tissue structure by producing spheres on microtiter plates [60, 61], which can be used to construct tumor cell spheroids and study the mechanisms of tumor cell proliferation, invasion, and metastasis [62, 63, 64]. However, the limitation of 3D cell culture models is that the central cells of larger spheres are prone to necrosis due to hypoxia and lack of culture medium [65]. In addition, 3D cell culture models have difficulty in fully reproducing the interactions between multiple cells in the body, and the challenges of complex workflows, reproducibility, and uniformity also limit its application [3, 66].

Animal models play a key role in new drug discovery and disease research, and are helpful for disease simulation, pathophysiology research, and preclinical evaluation of new drugs [67], such as in the research of lung cancer, pulmonary fibrosis, and other diseases [68]. However, due to species differences, high costs, animal welfare, and ethical factors [69], their performance in transforming into human treatment methods is poor [70, 71], making it difficult to accurately reproduce human diseases and drug reactions, thus hindering medical research and treatment development. Therefore, developing more accurate new models to overcome current challenges is crucial and urgent for promoting medical development and improving treatment outcomes.

### 2.2 The Birth and Construction of OoCs

**FIGURE 1:**
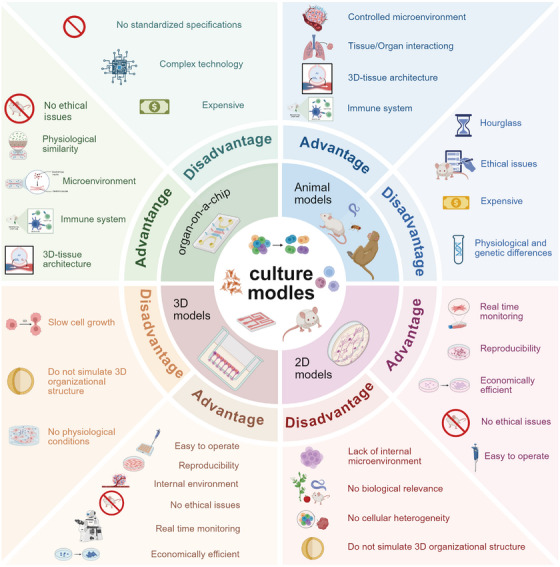
Comparative analysis of multiple cell culture models. The 3D microfluidic OoCs, which is superior to the existing models in multiple aspects, can be more conducive to new studies and discoveries related to human beings. Created with BioRender (www.biorender.com).

Because of the limitations of 2D and 3D cell culture models and animal models in simulating human physiology and pathology, OoC technology with its unique advantages has emerged [72, 73]. OoCs are a microfluidic cell culture device. It is designed to overcome the drawbacks of traditional culture models. It utilizes microchip manufacturing technology to construct a system containing continuously perfused chambers, where living cells are seeded and can mimic complex tissue structures and physiological functions [74, 75, 76]. By precisely controlling the microenvironment for cell growth, including factors like fluid flow, gas exchange, and intercellular interactions, cells are cultured in a way that is closer to the real in vivo state, thus providing a more effective tool for studying the physiological processes of human organs, disease mechanisms, and drug screening (Figure 1).

**FIGURE 2:**
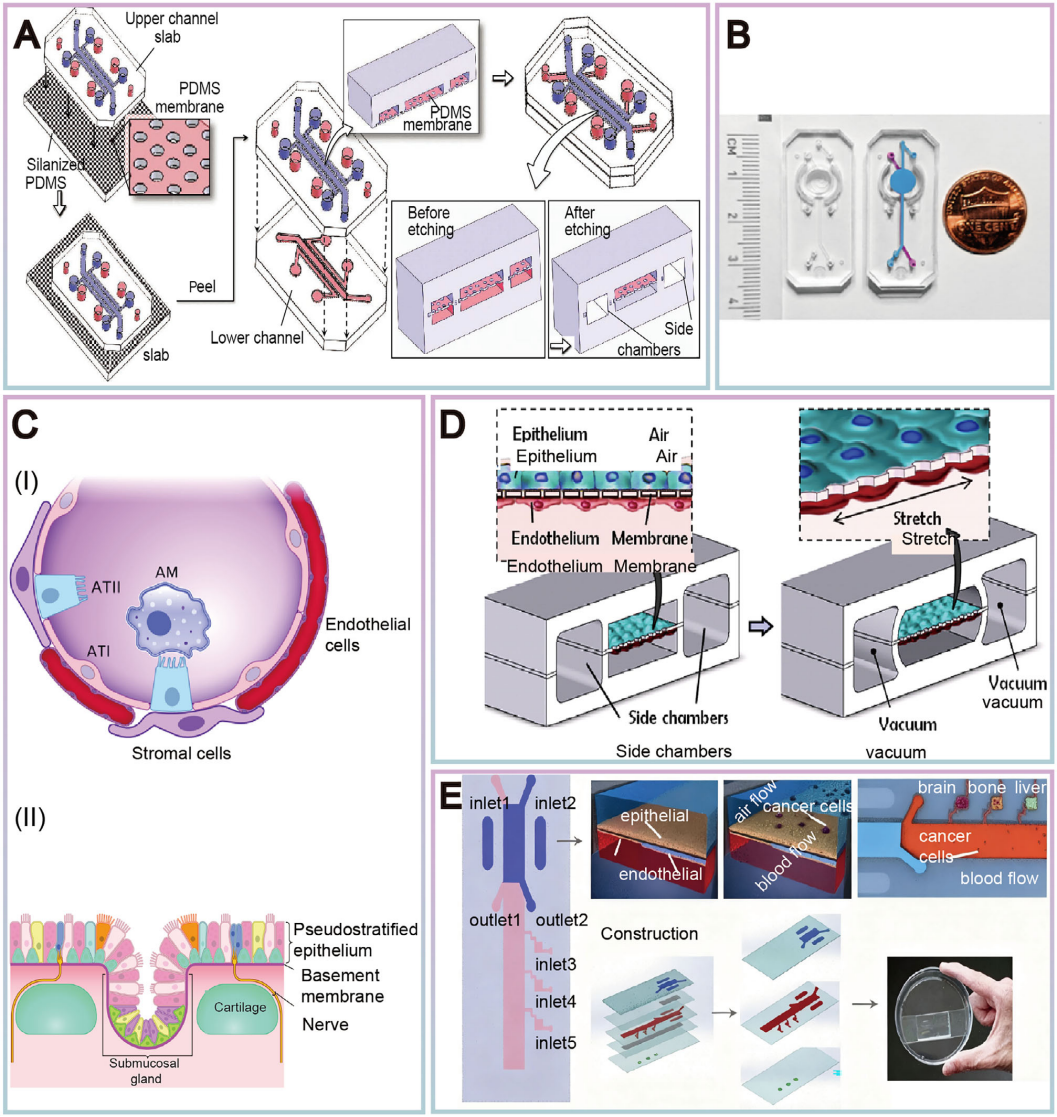
Design example diagram of lung‐on‐a‐chip. (A) Microfluidic lung chips are separated by porous PDMS membranes to create microchannels. Reproduced with permission from [12], copyright 2013 Nature Protocols. (B) Image of microfluidic chip. Reproduced with permission from [13], copyright 2021 Biomaterials. (C) (I) Human alveolar structure, including alveolar type I (ATI), alveolar type II (ATII), microvascular endothelial cells, stromal cells, and alveolar macrophages (AM). (II) The human airway is composed of many different cell types. Reproduced with permission from [14], copyright 2022 Physiology. (D) Lung chips simulate physiological respiratory movements by applying vacuum to the lateral chambers to generate mechanical stretching of PDMS membranes that form the alveolar capillary barrier. Reproduced with permission from [77], copyright 2010 Science. (E) Microfluidic chip simulates the four organ system of lung cancer cell metastasis to liver, bone, and brain. Reproduced with permission from [92], copyright 2016 ACS Applied Materials & Interfaces.

As a prominent representative of OoC technology, the lung‐on‐a‐chip possesses unique structures and functions. In 2010, Science magazine reported the pioneering reconstruction of the functional alveolar‐capillary interface of the human lung on OoCs, which could also simulate physiological respiratory movements [77]. This device features a microporous membrane situated between the two‐channel structures. The upper layer of the membrane is lined with human alveolar epithelial cells, while the bottom layer is lined with human pulmonary endothelial cells. Once the alveolar cells converge, the medium drawn in from the upper channel forms an air‐liquid interface with these alveolar cells (Figure 2) [78]. This study confirmed the feasibility of OoCs in reconstructing the complex and complete physiological responses of the lung organ. The lung‐on‐a‐chip micro‐device can simulate the key structures and functions of the human lung unit, with the induced and cultured endothelial and epithelial cells exhibiting complex organ‐level physiological functions unattainable through traditional in vitro culture. The lung‐on‐a‐chip reproduces the key structures, functions, and mechanical properties of the human alveolar‐capillary interface, which is also the basic functional unit of the lung [79]. After several years of rapid development, relying on microfluidic chips, researchers have initially developed numerous human OoC models, including those of the liver, lung, intestine, kidney, blood vessels, and heart [80]. In these microdevice chips, researchers have successfully cultured cells from various organs such as the kidney, brain, heart, and lung, and generated epithelial or endothelial tissues with differentiation capabilities [81, 82]. This marks the advancement of OoC technology from single‐organ research towards multi‐organ collaborative development. From a macroscopic perspective of technical application, OoC technology, with lung‐on‐a‐chip as a prominent representative, is now rapidly expanding its application fields, which have covered multiple key directions including cancer modeling [83], infectious disease research [84], bone‐related diseases [85] and exploration of neurodegenerative disease mechanisms [86]. The value of this technology is reflected not only in injecting new impetus into in‐depth disease research and effectively promoting scientific progress in related fields, but also in playing an important role in drug response evaluation. Meanwhile, it builds an efficient platform for the research, development and verification of potential therapeutic strategies, thereby providing crucial technical support for the innovative development of the biomedical field.

### 2.3 Existing Uses of OoCs in Diseases Investigations

**FIGURE 3:**
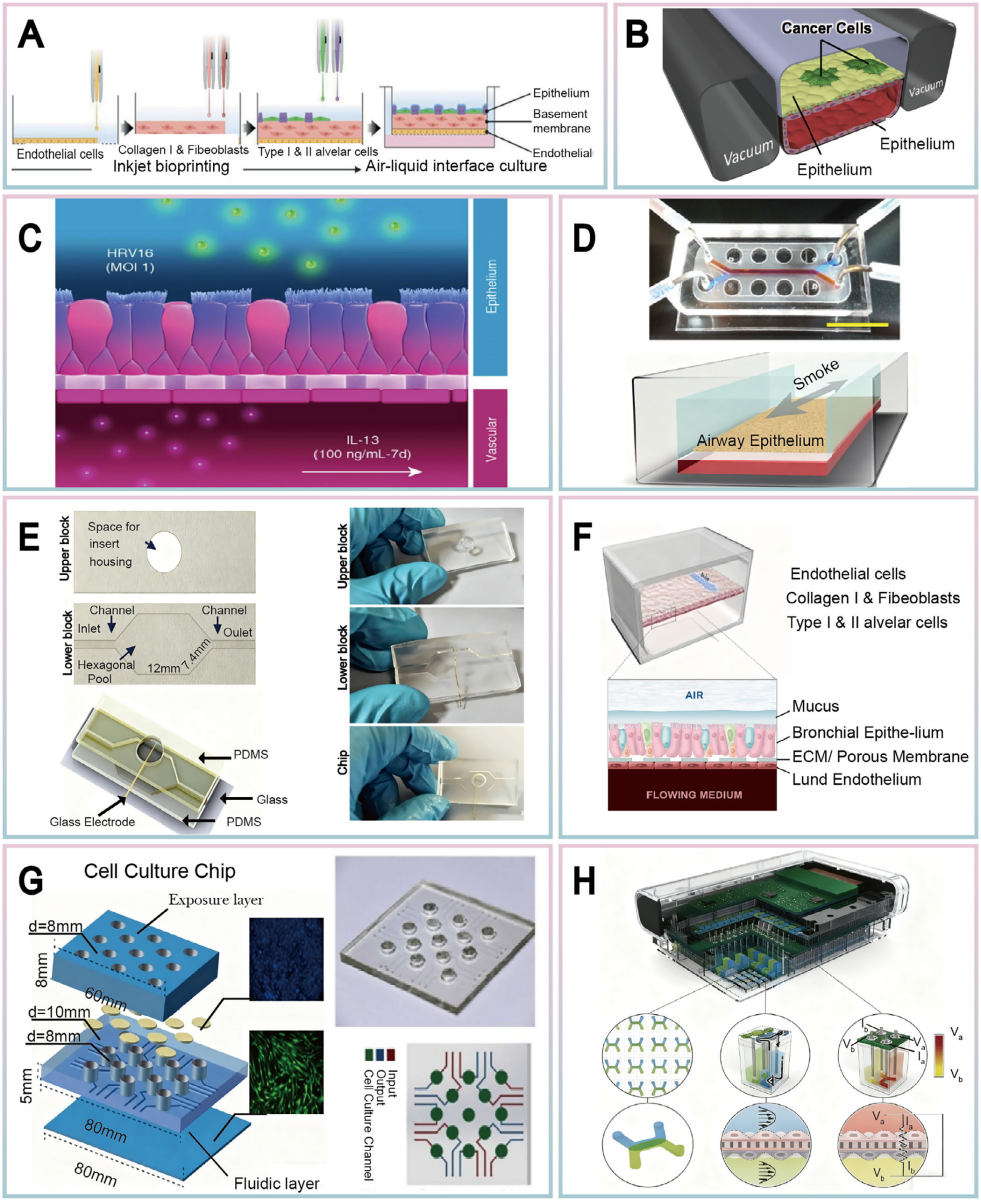
Application example diagram of OoCs in diseases. (A) 3D alveolar model for simulating influenza infection in the upper respiratory cortex, accurately simulate the process of pneumonia disease. Reproduced with permission from [88], copyright 2021 Advanced Science. (B) Non‐small cell lung cancer (NSCLC) model, simulating the unique growth pattern of alveoli in lung cancer chips. Reproduced with permission from [91], copyright 2017 Cell Reports. (C) The asthma epithelial cell model simulates asthma symptoms, indicating that airway lung chips can be used to evaluate the efficacy of immune response modulators. Reproduced with permission from Ref [94], copyright 2020 American Journal of Respiratory Cell and Molecular Biology. (D) Simulating the biological response of smoke inhalation through small airway cores can help deepen our understanding of the pathogenesis of COPD and asthma. Reproduced with permission from [95], copyright 2016 Cell Systems. (E) The cystic fibrosis (CF) chip model demonstrates that dynamic flow can enhance ciliary distribution and increase mucus volume, thereby promoting tissue differentiation in a short period of time. Reproduced with permission from [102], copyright 2023 ACS Biomaterials Science & Engineering. (F) (I) Simulating key features of CF airway using a cystic fibrosis chip based on the microenvironment of primary bronchial epithelial cells. (II) Health and CF chip vertical cross‐sectional immunofluorescence confocal microscopy images show: Ciliated cell surface β‐tubulin IV (green), basal cell surface CK5 (magenta); There are MUC5AC positive goblet cells (green) in the center; The bottom view (scale 10 μ m) shows the expression of CC‐10 (green). Reproduced with permission from [103], copyright 2022 Journal of Cystic Fibrosis. (G) On the MALIE microfluidic platform, it was found that PM2.5 promotes carcinogenesis by affecting ErbB family members and their downstream signaling pathways. Reproduced with permission from [108], copyright 2022 Ecotoxicology and Environmental Safety. (H) A novel high‐throughput OoC platform that integrates programmable fluid flow and real‐time sensing technology for complex tissue model research in drug development. Reproduced with permission from [111], copyright 2021 Lab on a Chip.

Pneumonia, particularly viral pneumonia, represents a significant global health challenge [87]. Taking the research by Jung et al. [88] exemplifies the state‐of‐the‐art in this field. Utilizing inkjet printing technology, they constructed a high‐resolution 3D alveolar chip incorporating influenza virus‐sensitive NCI‐H441 (alveolar epithelial type II cells) and MRC‐5 (lung fibroblast) cell lines (Figure 3A). Following the introduction of influenza A virus (H1N1) into the airway compartment, the model successfully recapitulated virus‐induced cytopathic effects and inflammatory responses, demonstrating strong correlation with clinical observations. This system enables real‐time, in‐situ investigation of virus–host interactions, thereby providing a robust platform for elucidating viral replication mechanisms and evaluating the efficacy of antiviral therapeutics.

Lung cancer remains one of the most prevalent and lethal malignancies worldwide. Current clinical management strategies primarily include surgical resection, chemotherapy, radiotherapy, and immunotherapy [89]. However, therapeutic failure is frequently associated with the complexity of the tumor microenvironment and the development of drug resistance in tumor cells, representing major challenges in clinical management [90]. To overcome the limitations of conventional models in replicating human tumor biology, OoC technology has emerged as a highly biomimetic in vitro platform for lung cancer research. For instance, Hassell et al. [91] developed a lung cancer‐on‐chip model specifically designed to simulate the pathological processes of non‐small cell lung cancer (Figure 3B). This model established a tumor microenvironment incorporating both cancer cells and stromal components, successfully recapitulating key pathological features including tumor growth, dormancy, and response to tyrosine kinase inhibitor treatment. Real‐time monitoring revealed that tumor cells within the chip not only formed tissue‐like three‐dimensional structures but also exhibited proliferation dynamics and drug responses that closely mirrored clinical observations, providing a powerful tool for investigating lung cancer progression and drug resistance mechanisms. Furthermore, the multi‐organ chip model developed by Xu et al. [92] enabled the simulation of lung cancer cell invasion and distant metastasis processes. This platform provides crucial insights into the mechanisms underlying advanced lung cancer metastasis and the distribution effects of drugs across different organs, significantly expanding the application prospects of OoC technology in cancer metastasis research.

Asthma is a common disease caused by chronic inflammation of the lower respiratory tract [93]. Nawroth JC et al. [94] developed an asthmatic epithelial model using an airway‐on‐chip platform (Figure 3C). By culturing human primary airway epithelial cells (hAECs) and human lung microvascular endothelial cells (hMVECs) in the Airway Lung‐Chip, the model demonstrated how viral infections trigger abnormal immune cell recruitment and epithelial responses, providing a platform to investigate mechanisms of asthma exacerbation and potential intervention strategies. Benam KH et al. [95] demonstrated a small airway core that “inhales” and sucks out the entire cigarette smoke from a chip (Figure 3D). By simulating the biological response of smoke inhalation, researchers were able to observe cilia micro pathological changes, chronic obstructive pulmonary disease (COPD) specific molecular feature expression, and epithelial response changes in the cells on the chip to the smoke produced by electronic cigarettes. These simulation experiments contribute to a deeper understanding of the pathogenesis of COPD and asthma, providing important theoretical basis for the development of targeted treatment methods.

COPD is a major cause of global morbidity, mortality, and healthcare utilization. COPD is caused by exposure to inhaled harmful particles, the accumulation of airway or lung gases, and inflammatory cells [96, 97]. Its pathological feature is mainly the irreversible airflow limitation resulting from peripheral airway inflammation [98]. Small airways, also known as bronchioles, are the tiny air passages in the lungs. They play a crucial role in transporting air into the lungs and excreting secretions. Therefore, the health of small airways is vital for the function of the entire respiratory system [99]. The air–liquid interface established by the small airway‐on‐a‐chip enables the complete differentiation of small airway epithelial cells. Moreover, adding immune cells into the liquid flow channel can also clarify the important role of epithelial cells in immune balance. The lung small airway‐on‐a‐chip model developed by Benam et al. [100] represents an important technological advancement. This model contains differentiated mucociliary bronchiolar epithelium and underlying microvascular endothelium experiencing liquid flow. By exposing the epithelium to interleukin 13 (IL‐13), it has successfully reconstructed pathological features such as goblet cell hyperplasia, excessive cytokine secretion, and reduced ciliary function in asthma patients, and can accurately simulate the complex processes of inflammatory lung diseases, including diseases like asthma and COPD. The observation of COPD‐specific molecular signatures and pathological changes on the chip directly demonstrates the central role of environmental toxins in driving disease pathogenesis.

Pulmonary fibrosis and cystic fibrosis (CF) are diseases characterized by abnormal ECM deposition or mucus dysfunction, respectively [101]. In the research of Claudia Mazio et al. [102], they successfully constructed a CF chip model (Figure 3E), which has the ability to simulate human bronchial epithelial cell culture in vitro and achieve cell differentiation and processing quickly. This research demonstrated that dynamic flow enhances cilia distribution and increases mucus volume, thereby promoting tissue differentiation within a short timeframe. Utilizing this model, they observed that the corrector agent VX‐809 effectively reduced mucus thickness and viscosity. In a complementary study, Plebani R et al. [103] constructed a CF airway chip using patient‐derived primary cells (Figure 3F), which accurately replicated core pathological features of patient airways, including IL‐8 hypersecretion, massive neutrophil infiltration, and the creation of a favorable environment for *Pseudomonas aeruginosa* growth. This model not only elucidates the pathophysiological process of CF but also serves as a platform for therapeutic efficacy evaluation. The CF chip reproduces key features of airway dysfunction in CF patients, providing an important tool for the study of CF pathophysiology. Figure 3 showcases representative applications of OoC technology, while Table 1 presents various physiological and pathological models. Regarding cellular sources, cancer models predominantly utilize tumor cell lines or primary tumor cells; airway‐related models typically employ primary or immortalized airway epithelial cells; organoid models rely on adult or pluripotent stem cells; whereas microfluidic cell culture chip models demonstrate broader cellular source applicability.

**TABLE 1:** Comparison of key indicators in various physiological and pathological models.

Model	Materials	Physiological relevance	Cell source and ethics	Immune research	Mechanical force simulation	Co‐culture	Limitations	Ref.
Cancer	PDMS	Relatively high	Tumor cell lines/primary tumor cells, favorable ethical profile	Studies tumor immune microenvironment	Simulates tumor tissue stiffness	Supported	Difficult to replicate tumor heterogeneity; inadequate simulation of metastasis	[91]
Airway epithelial	PDMS	High	Primary/immortalized airway epithelial cells, favorable ethical profile	Studies airway immune responses	Simulates respiratory stretch, mucociliary function	Supported	Limited simulation of neural regulation; cell degeneration in long‐term culture	[88, 95, 103]
Organoid‐related	Matrigel	Relatively high	Adult/pluripotent stem cells, relatively favorable ethical profile	Simple immune components	Simulates mechanical microenvironment within tissue	Supported	Lacks complete system; poor batch‐to‐batch consistency	[94]
Cell culture chip	PDMS	Moderate	Various cell lines/primary cells, favorable ethical profile	Customizable for immune experiments	Simulates localized fluid shear stress	Supported	Inadequate simulation of multi‐cell/tissue coordination	[108]
Microfluidic airway	PDMS	High	Primary airway cells/cell lines, favorable ethical profile	Correlates immunity and fluid dynamics	Simulates airway hydrodynamic environment	Supported	Complex chip design, limited throughput, long‐term stability needs improvement	[102]

The successful application of OoC technology in disease research has established it as a “bridging technology” connecting preclinical studies and clinical trials, demonstrating significant translational potential. This potential manifests in several key aspects: Through multi‐omics analysis (e.g., proteomics, metabolomics) of micro‐samples within the chips, novel biomarkers for disease diagnosis or treatment response can be discovered [104]. Personalized lung chips constructed using patient‐derived cells enable optimal treatment selection for specific individuals, advancing precision medicine. With their high predictive capacity, this technology not only accelerates new drug screening but also facilitates testing of new indications for approved drugs in rare respiratory diseases (drug repositioning). Furthermore, as the technology matures and data accumulates, lung chips are expected to partially replace animal models, providing more reliable evidence for preclinical drug evaluation [105]. However, comprehensive translation of this technology faces several challenges: The primary task involves standardizing chip designs, cell sources, and culture conditions across different laboratories, establishing validated protocols through correlation with clinical data [106]. Second, model complexity needs enhancement through integration of adaptive immunity, innervation, and gut–lung axis components to better mimic human physiology. Concurrently, reducing manufacturing costs and developing automated high‐throughput platforms are essential for industrial‐scale applications [107]. Ultimately, regulatory acceptance of OoCs data for drug approval requires collaborative efforts from academia, industry, clinicians, and regulators, supported by robust evidence generation.

### 2.4 The Current Application of OoCs in Drug Development

OoCs also plays a crucial role in evaluating treatment effects and drug screening [109, 110]. Microfluidic OoC technology has shown great potential in new drug research and development due to its advantages such as high throughput, automation, real‐time multi‐index monitoring, and accurate simulation of relevant in vivo microenvironments. This technology can predict the efficacy and toxicity of drugs more quickly and accurately, providing a new direction for drug screening [33, 111].

Drug development is a complex and time‐consuming process. Adverse drug reactions are one of the major challenges in drug development and have become a major burden on global public health services [112]. The pharmacokinetics–pharmacodynamics (PK–PD) model, a mathematical model based on human physiology, plays a crucial role in the drug development process. This model can simulate the processes of absorption, distribution, metabolism, and excretion (ADME) of drugs in the body, which are the two main principles determining the relationship between dosage and response [113]. The newly developed microscale cell culture analog (mCCA) is a physical representation of this PK–PD model. This model can simulate the physiological environment inside the human body and provides a physical basis for the PK–PD model in the drug development process. By simulating the microenvironment within the human body, mCCA can help researchers better understand and predict the behavior of drugs in the body. Sung JH et al. [114] designed a three‐compartment mCCA containing three cell lines, namely liver, tumor, and bone marrow, to test the toxicity of the anticancer drug 5‐fluorouracil (5‐FU). The results were analyzed using the PK–PD model of the device and compared with those under static conditions. Each cell type responded differently to 5‐FU, and the responses in the microfluidic environment were different from those in the static environment. By replicating the interactions among organs in vivo, it was demonstrated that the mCCA model has the potential to test the toxicity of drugs that is dependent on drug metabolism.

The evaluation of drug toxicity and efficacy is crucial for drug development [115], and accurate dosage is related to treatment effectiveness. Improper dosage can cause side effects or poor efficacy. Although animal testing is an important part of drug safety evaluation, it is prone to unnecessary rejection of candidate drugs and cannot accurately predict human toxicity [116]. And animal testing also has other drawbacks. There are also problems such as being time‐consuming, costly, and difficult to accurately predict human adverse reactions [117]. In order to obtain more reliable results, Huhdd et al. [79] developed a “lung‐on‐a‐chip” to simulate human lung function. Researchers used this lung chip model to study the toxicity induced by IL‐2 and found that physiological respiratory movement plays a key role in IL‐2‐induced tissue damage, confirming that lung chips can evaluate the expected and unexpected toxicity of drugs. Yang X et al. [118] used nano‐electrospinning technology with polylactic acid‐hydroxyacetic acid copolymers to fabricate ultra‐thin porous membranes to simulate alveolar respiratory membranes. When they evaluated gefitinib by culturing relevant cells on both sides of the membrane, they found that A549 cells can cause endothelial cell apoptosis, death, and tumor invasion. Frost TS et al. [119] established a small airway chip. They connected the jet spray to simulate the blood–gas barrier of the human small airway. Then, they measured the permeability of various compounds, constructed a concentration‐time diagram, extracted pharmacokinetic (PK) parameters, and screened the ability of compounds to pass the airway epithelial‐endothelial barrier. All these efforts helped to develop a new inhalation therapy. Jain A et al. [120] used lipopolysaccharide (LPS) infection to indirectly stimulate intravascular thrombus formation in the upper‐layer human alveolar epithelial primary cells in whole‐blood perfusion alveolar chips. This model was utilized to assess the inhibitory impact of protease‐activated receptor 1 (PAR‐1) antagonists on endothelial activation and thrombus formation. As an in vitro model of pulmonary microvascular thrombosis, this alveolar chip can quickly screen antithrombotic drugs, thus demonstrating the potential of organ chips in drug screening and providing a basis for their widespread application.

### 2.5 Clinical Translation Applications of OoCs

Leveraging highly biomimetic human physiological models, OoC technology is increasingly emerging as a pivotal bridge connecting in vitro research with clinical trials, demonstrating significant translational value across multiple cutting‐edge therapeutic areas [121]. In preclinical research, this technology not only serves to initially validate the efficacy and safety of candidate drugs but also generates human‐specific data that can potentially replace certain animal studies, thereby providing critical supporting evidence for innovative therapies.

In the study of inflammatory lung diseases, the preclinical research on Azeliragon—a small‐molecule anti‐inflammatory drug that acts by blocking the RAGE receptor—fully demonstrates the translational value of OoC technology. Using a human lung chip model, researchers successfully simulated and validated the drug's ability to inhibit the cytokine storm at the alveolar‐capillary interface [100]. Based on the high human relevance and mechanistic depth demonstrated in this study, along with reliable data obtained from subsequent larger‐scale validation [122], the drug ultimately gained regulatory approval to bypass certain conventional animal studies and proceed directly into Phase II clinical trials. This made it the first new drug worldwide to receive FDA approval for clinical trials based primarily on lung chip data. In the field of neurological disease research, a candidate drug developed by Sanofi utilized an iPSC‐derived motor neuron chip model to generate key pharmacological data [123]. These data were accepted by the FDA and supported the Investigational New Drug application, highlighting the unique value of OoC technology in developing therapies for complex neurological disorders. In cardiovascular research, the domestically developed candidate drug HRS‐1893—a cardiac myosin inhibitor—underwent screening and functional validation of hundreds of compounds using a heart‐on‐a‐chip platform before entering clinical studies [124]. This approach rapidly identified the optimal candidate molecule for improving myocardial contraction and relaxation, significantly accelerating the Investigational New Drug approval process and making it the first innovative drug in China to enter clinical trials based on heart chip data. Regarding drug safety evaluation, studies have shown that liver chips can accurately identify 87% of hepatotoxic drugs, significantly outperforming traditional animal models (50%–65%) [125]. Furthermore, liver chips developed by Emulate have demonstrated the ability to detect hepatotoxicity signals that are often missed in conventional animal studies [126], providing a more reliable predictive tool for preclinical safety assessment.

The cases presented in Table 2 vividly demonstrate the crucial role of organ chips in drug development and their growing status in regulatory science. As an effective supplement to animal testing, this chip technology holds significant potential to substantially reduce drug development costs while providing more predictive human‐relevant data to support regulatory decision‐making. With regulatory agencies like the FDA progressively promoting the adoption and standardization of alternative methods to animal testing, emerging technologies such as OoCs are evolving from “alternative approaches” toward becoming “regulatory standards,” marking the emergence of a new drug development paradigm centered on human biology [127].

**TABLE 2:** Representative drug cases advanced into clinical stages promoted by OoC technology.

Disease area	Drug/Candidate	Development stage	Pathway & mechanism of action	OoC model application & value	Ref.
Inflammatory lung disease	Azeliragon (TTP488)	Phase II clinical trials	Receptor for Advanced Glycation End‐products (RAGE) antagonist; reduces inflammatory response by inhibiting the RAGE signaling pathway	The world's first new drug approved by the FDA for clinical trials based on lung‐on‐a‐Chip data. Its anti‐inflammatory mechanism was validated using a human alveolar‐capillary chip model, with data directly supporting the IND application	[100, 122]
Rare neurological disease	CenoFi candidate drug	IND stage	Targets a specific antigen for autoimmune demyelinating disease	Key pharmacological data obtained from a motor neuron chip model based on iPSC differentiation were recognized by the FDA, supporting its IND application	[123]
Cardiovascular disease	HRS‐1893	Phase I clinical trials	Cardiac myosin ATPase inhibitor; improves myocardial diastolic function	The first innovative drug in China to enter clinical trials based on heart‐on‐a‐chip data, having undergone efficient screening and optimization on the chip platform	[124]
Drug safety evaluation	Multiple candidate drugs	Preclinical stage	Hepatotoxicity of drug metabolites	The liver‐on‐a‐chip correctly identified 87% of hepatotoxic drugs, significantly outperforming traditional animal models (50%–65%)	[125, 126]

## 3 Omics Technologies' Use in OoC Models

**FIGURE 4:**
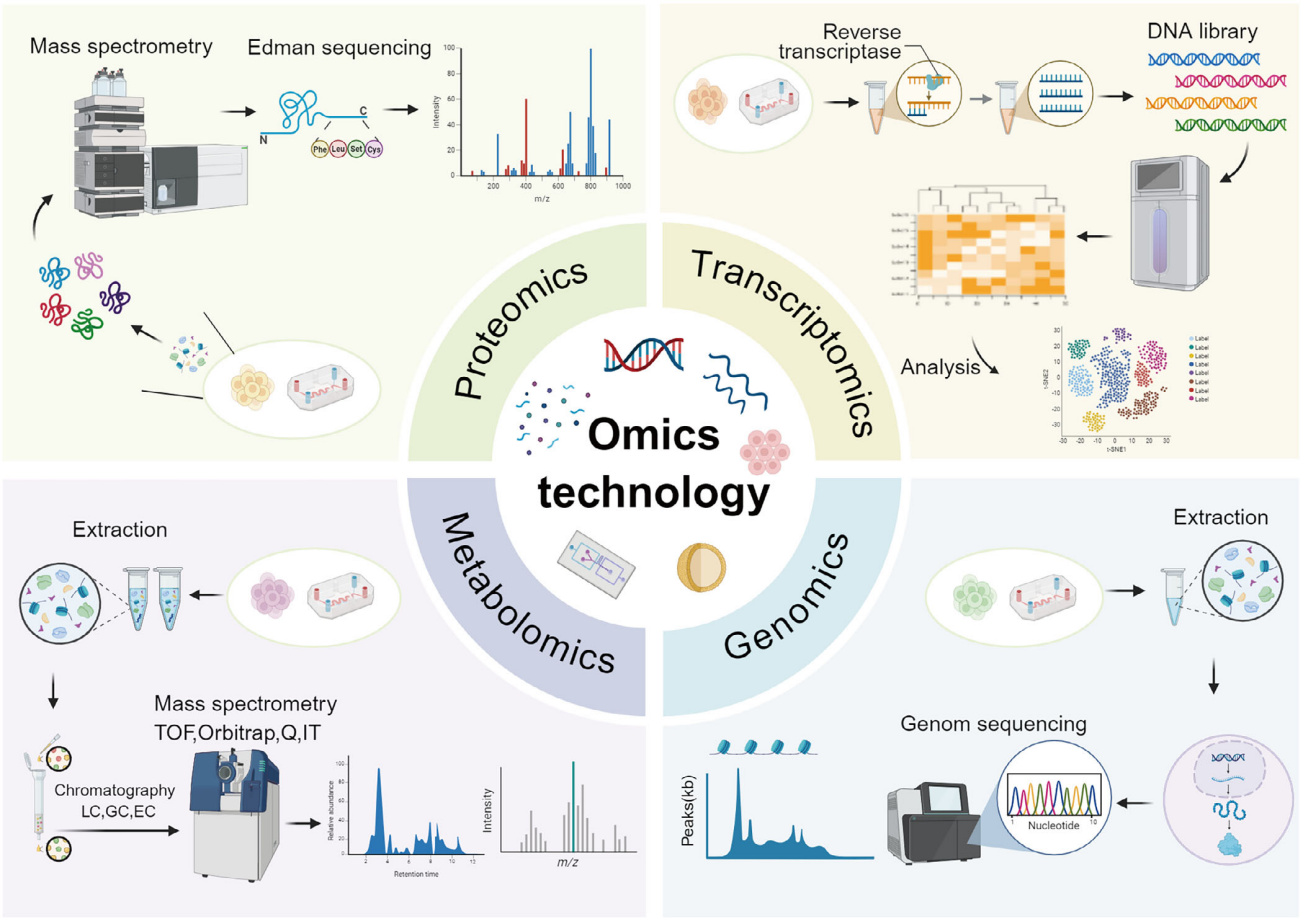
Classification of omics techniques includes proteomics, transcriptomics, metabolomics, and genomics. These different omics fields analyze the molecular mechanisms of life activities from distinct perspectives. Proteomics focuses on all the proteins expressed in cells, tissues, or organisms and conducts in‐depth analyses of protein expression levels, post‐translational modifications, and interaction networks. Transcriptomics uses high‐throughput sequencing and other technologies to comprehensively analyze transcript changes under different physiological and pathological conditions. Metabolomics primarily studies small molecule metabolites in living organisms. Genomics focuses on the entire genetic sequence of organisms at the fundamental level of omics research. Created with BioRender (www.biorender.com).

Multi‐omics technology integrates high‐throughput analytical methods such as genomics, transcriptomics, single‐cell transcriptomics, proteomics, metabolomics, and spatial transcriptomics. It reveals the molecular basis of life activities from a systematic perspective and has become a key driver in advancing human disease research [128, 129]. Various omics technologies focus on biomolecules at different levels, forming a complete analytical chain from genetic information to functional execution (Figure 4). Genomics takes the entire gene sequence of an organism as its research object. For instance, the Human Genome Project mapped a complete genomic profile, laying a fundamental framework for understanding the laws of genetic trait inheritance and the genetic background of diseases. Transcriptomics focuses on gene transcription products; as a crucial link connecting genomic information and protein function, it plays an irreplaceable role in revealing the dynamic regulation of gene expression [130]. Proteomics further analyzes the expression levels, modification states, and interaction networks of proteins, directly reflecting the execution mechanisms of life activities and regulating cell growth, differentiation, and stress responses [131]. Metabolomics focuses on the composition and changes of small‐molecule metabolites in cells. As the end products of metabolic activities, their dynamics can intuitively reflect the functional output of the organism under physiological or pathological conditions [132, 133].

**FIGURE 5:**
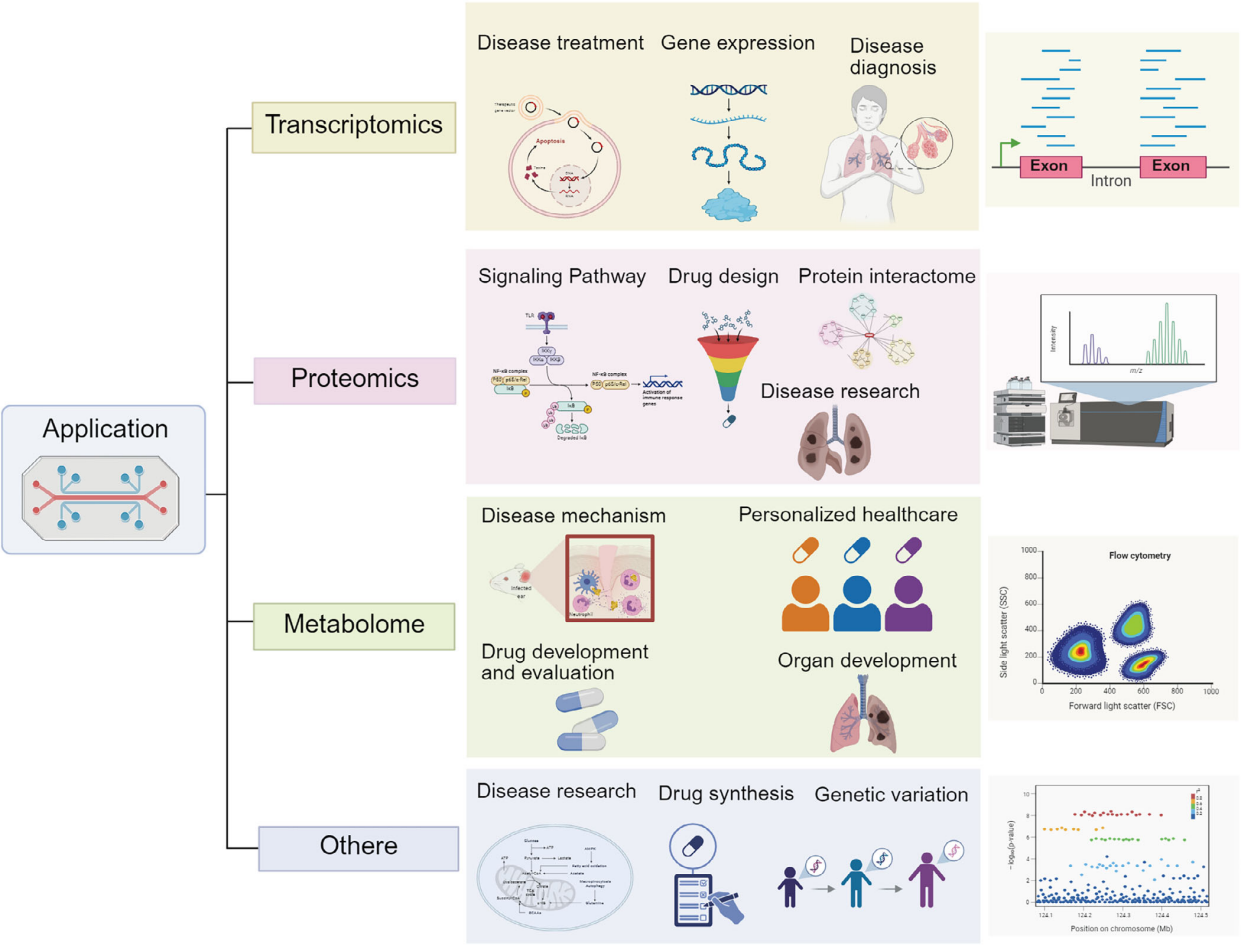
The multifaceted application of omics technology in disease exploration and drug research. In terms of disease exploration, proteomics can analyze protein expression, modification, and interaction networks, facilitating the discovery of disease‐related protein biomarkers; transcriptomics reveals changes in gene expression and regulatory networks during the occurrence and development of diseases by detecting gene transcripts; metabolomics studies small molecule metabolites in living organisms, revealing the correlation between cellular metabolic status and diseases. In the field of drug research, these omics technologies can provide key information for drug design, screening, and efficacy evaluation from different levels. Created with BioRender (www.biorender.com).

The development of the aforementioned omics technologies has laid a critical foundation for conducting systematic molecular measurements on OoC platforms, which are highly biomimetic. OoC technology, with its precise control over the microenvironment, can simulate the physiological structure and function of human organs (Figure 5). Different omics methods complement each other on this platform: Proteomics analyzes molecular interactions at the “functional execution” level, transcriptomics captures gene expression changes at the “dynamic regulation” level, and metabolomics reflects metabolic states at the “terminal physiological effect” level. The integrated application of these three omics approaches enables the construction of molecular event networks with clearer causal relationships through cross‐validation. Particularly in lung disease research, this integration not only aids in deepening the understanding of disease mechanisms but also accelerates the drug development process. To more clearly illustrate the specific application scenarios of omics technologies in OoC models, representative research cases are listed in Table 3 below, providing references for subsequent related studies.

**TABLE 3:** Representative examples of the application of omics technology in OoC models.

Omics technology	Disease	Channel	Materials	Cell sources	Applications	Publication time	Ref.
Proteomics	Lung cancer	Three	PDMS	Human lung cancer cell line PC9	Drug resistance	2020	[47]
	Hepatotoxicity	Two	—	Primary human liver related cells	Liver toxicity mechanism	2023	[136]
	Lung cancer	One	PDMS	Lung cancer organoid (LCO)	Drug efficacy and drug toxicity	2019	[137]
Transcriptomics	Pneumonia	Two	PDMS	Human bronchial epithelial (HBE) cells	Mechanism of respiratory inflammation	2024	[138]
	Pneumonia	Two	PDMS	Alveolar epithelial cells, vascular endothelial cells, alveolar macrophages, and peripheral blood mononuclear cells (PBMCs)	Pathogenesis	2024	[139]
	Lung cancer	Two	PDMS	Human bronchial epithelial (HBE) cells	Carcinogenic effects induced by PM2.5	2022	[108]
	Lung cancer	Two	PDMS	Lung cancer cells, HFL‐1 fibroblasts, and L02 liver cells	Molecular mechanism and Drug efficacy	2021	[140]
	Depression	Two	PDMS	Human‐induced PSC (hiPSC)	Organ metabolism toxicity process, drug toxicity	2020	[141]
	IAV	Two	PDMS	Human primary alveolar epithelial cells (AECs), human pulmonary microvascular endothelial cells (HPMECs)	Evaluate the targeted efficacy of CRISPR RNA therapy	2025	[142]
Metabolomics	Pneumonia	Two	PDMS	Human bronchial epithelial (HBE) cells	Mechanism of respiratory inflammation	2019	[143]
	Hepatotoxicity	One	PDMS	Primary human liver related cells	Hepatotoxicity monitoring	2016	[144]
Other	Lung cancer	—	PDMS	Small airway epithelial cells, vascular endothelial cells, stromal cells, and macrophages	Pulmonary toxicity, drug toxicity	2025	[145]
	Intestinal diseases	Two	PDMS	Human Caco2 intestinal epithelial cells, human intestinal microvascular endothelial cells	Research and development of intestinal related therapies	2019	[146]

### 3.1 Potential Application of Proteomics in the Study of Diseases

Proteomics occupies an extremely important position and has extensive applications in the research of OoC models for diseases. The concept was first proposed by Wilkins et al. in 1995 [134], referring to the complete set of proteins expressed by a genome, cell, or tissue under specific conditions. Its core lies in the systematic analysis of the composition of proteins, changes in their expression levels, and modification statuses, thereby providing key evidence for uncovering disease mechanisms, discovering potential biomarkers, and identifying drug targets.

In drug development, proteomics enables precise delineation of drug‐induced alterations in cellular protein expression, thereby providing molecular‐level evidence for both efficacy assessment and toxicity prediction [135]. This advantage is fully demonstrated in research paradigms integrating OoCs with multi‐omics approaches. In the field of cancer research, Xu M et al. [47] successfully modeled the complete process of lung cancer metastasis from the primary site to the brain using a microfluidic multi‐organ chip platform. Proteomic analysis revealed significant upregulation of key enzymes in the glutathione metabolic pathway—including GPX4, RRM2, and GCLC—in the brain‐tropic subpopulation PC9‐Br cells, unveiling the significant role of disrupted redox homeostasis in tumor metastasis and the acquisition of multidrug resistance. Similarly, in toxicology research, Goralski et al. [136] established a multi‐omics analytical workflow for minimal samples using a human liver chip, systematically elucidating the broad impact of the chemical warfare agent VX on hepatic energy metabolism. By integrating proteomic, metabolomic, and transcriptomic data, the study not only confirmed VX‐induced reprogramming of core metabolic pathways, such as glycolysis and the tricarboxylic acid cycle, but also identified significant alterations in mitochondrial function‐related proteins including GAPDH and CAT, providing novel perspectives for understanding VX hepatotoxicity mechanisms. In the context of respiratory disease drug development, proteomics likewise demonstrates considerable value. Jung et al. [137] developed an integrated microfluidic platform that enables both 3D lung cancer organoid culture and drug evaluation. Their study found that while cisplatin and etoposide induced concentration‐dependent apoptosis, resistant cell populations persisted in the organoid core regions. This model provides an ideal platform for subsequent proteomic analysis, which through systematic characterization of protein expression, differences between drug‐sensitive and resistant regions could reveal critical resistance targets, thereby supplying molecular evidence for developing targeted therapeutic strategies. Collectively, these studies exemplify the central role of proteomics in deciphering disease mechanisms and guiding drug design, providing crucial technological support for advancing precision medicine.

In conclusion, the application of proteomics technology and microfluidic OoC technology in the new generation of OoC models provides powerful tools for the research of diseases and drug development, which is conducive to accelerating the process of new drug discovery and development.

### 3.2 Transcriptomic Analysis in Disease Research

The application of transcriptomics in OoC models of diseases provides a unique and powerful platform for in‐depth exploration of the molecular mechanisms of diseases. It mainly focuses on analyzing changes in gene expression. Through comprehensive detection and analysis of transcripts in cells under different physiological and pathological conditions, it accurately reveals the molecular mechanisms during the occurrence and development of diseases and provides crucial evidence for evaluating drug efficacy and predicting drug side effects.

In environmental toxicology and inflammation research, Li Z et al. [138] developed a three‐layer PDMS chip integrating gas concentration gradient generation and liquid perfusion systems to simulate human bronchial epithelial exposure to PM2.5. RNA‐Seq analysis identified 943 differentially expressed genes (725 upregulated, 218 downregulated), with GO and KEGG enrichment analyses revealing predominant involvement in inflammatory and stress response pathways, particularly MAPK signaling. The study further demonstrated that dexamethasone intervention effectively reversed PM2.5‐induced gene expression alterations, providing new mechanistic insights into PM2.5‐driven respiratory inflammation and anti‐therapeutic development. In parallel, for viral infection and immune response studies, Wang P et al. [139] established a multicellular human lung chip incorporating alveolar epithelium, vascular endothelium, alveolar macrophages, and peripheral blood mononuclear cells to model influenza A virus infection. Single‐cell RNA sequencing revealed that viral infection primarily triggered epithelial inflammatory responses that propagated to endothelial cells, causing barrier dysfunction. Immune profiling highlighted the central role of B cells and CD4+ T cells in immune regulation, showing marked activation of type I interferon signaling and cytokine/chemokine networks, with multiple intercellular communication‐related genes significantly upregulated. Collectively, these chip‐based models provide sophisticated platforms for investigating environmental toxin‐induced pathogenesis and viral immune mechanisms, advancing drug discovery and therapeutic strategy development.

In the field of multi‐omics integration and carcinogenesis mechanism exploration, Zheng L et al. [108] developed a portable, fully integrated lung‐on‐a‐chip system capable of culturing HBE cells at the air‐liquid interface under PM2.5 exposure. Integrated whole‐genome DNA methylation and transcriptome analysis revealed that PM2.5 may promote cellular malignant transformation through regulation of ErbB family receptors and their downstream signaling pathways, providing new insights into epigenetic mechanisms of PM2.5‐related respiratory diseases and targeted therapy development. In tumor metastasis and drug screening research, Zheng L et al. [140] constructed a 3D‐CMOM platform with precise oxygen control to investigate hypoxia‐induced liver metastasis mechanisms in lung cancer. RNA‐seq analysis identified 887 differentially expressed genes in A549 cells under hypoxia, primarily involved in epithelial‐mesenchymal transition and tumor metastasis pathways. Mechanistic studies demonstrated enhanced metastatic propensity through A549‐HFL‐1 cell interactions in hypoxic microenvironments, while drug screening confirmed compound SYP‐5's significant anti‐cancer activity with low hepatotoxicity under hypoxia, highlighting the platform's potential for cancer research and drug discovery. In viral tropism mechanism investigation. Han Y et al. [141] created a hiPSC‐derived multi‐organoid chip system integrating liver and heart organoids to evaluate clomipramine's metabolism‐dependent cardiotoxicity. Transcriptome analysis confirmed high expression of CYP450 enzymes in liver organoids, demonstrating substantial metabolic capacity. The study revealed that active metabolites generated through hepatic metabolism reduced viability and impaired contractile function in co‐cultured cardiac organoids, successfully modeling in vivo drug metabolism‐toxicity processes and providing a novel platform for drug safety assessment. Most notably, Man et al. [142] pioneered the integration of CRISPR‐Cas13 gene editing technology with human alveolar chips, establishing a preclinical evaluation system for influenza A virus (IAV) CRISPR RNA therapeutics. The study designed broad‐spectrum crRNAs targeting viral PB1 protein and demonstrated, using alveolar chips that accurately replicate alveolar physiological microenvironments (including air‐liquid interface, respiratory mechanics, and immune cell recruitment), simultaneous suppression of viral replication and host inflammatory responses. Safety assessment via transcriptomic profiling (RNA‐seq) ruled out Cas13d off‐target effects, ultimately establishing a “CRISPR RNA therapy + human organ chip” paradigm that transcends the limitations of animal models for preclinical evaluation.

The analysis of gene expression changes by transcriptomics provides important insights into disease occurrence and development. As shown in Figure 5, transcriptomics plays an important role in exploring disease mechanisms and evaluating drug efficacy. With the advancement of cutting‐edge technologies such as single‐cell transcriptomics and spatial transcriptomics, this interdisciplinary research strategy integrating engineering platforms with multi‐omics analysis demonstrates substantial potential in the era of precision medicine, while paving new pathways for promoting the integration and clinical translation of multi‐omics data.

### 3.3 Metabolomics Analysis in Disease Research

Metabolomics measures the end products of cellular metabolic activities, most directly reflecting the physiological and pathological states of cells. Particularly in multi‐organ chips, it can impartially reveal perturbations to metabolic pathways induced by drugs or toxins‐a distinct advantage that other omics approaches cannot readily replace [147, 148].

In relevant studies, Wang X et al. [143] established a groundbreaking multi‐organ assessment platform by integrating a “human‐on‐a‐chip” system comprising seven interacting microphysiological systems (including brain, pancreas, liver, and lung). Through mass spectrometry analysis, they characterized tolcapone's metabolic profile, identifying 12 metabolites (including three newly discovered compounds). Metabolomic analysis of the brain chip revealed drug‐induced alterations in 18 key biomarkers, particularly disruptions in tryptophan/phenylalanine metabolism pathways, suggesting potential impacts on neurotransmitter synthesis. Concurrently identified abnormalities in glycerophospholipid and energy metabolism provided novel insights into the drug's mechanism of action. This research established an integrated evaluation platform combining drug metabolism studies with multi‐organ chip technology, creating a new paradigm for drug safety assessment. Similarly representative is the technological breakthrough in real‐time monitoring by Riahi et al. [144]. The team developed an innovative automated microfluidic bead‐based electrochemical immunosensor integrated with a liver‐on‐a‐chip bioreactor, enabling continuous dynamic monitoring of proteins secreted by hepatocytes. In an acetaminophen‐induced hepatotoxicity model, the platform successfully achieved precise monitoring of transferrin and albumin secretion from human primary hepatocytes over five consecutive days. The sensor demonstrated exceptional performance with an online detection sensitivity of 0.03 ng/mL, significantly surpassing traditional ELISA methods, while maintaining reusability through bead replacement. This technological advancement provides an efficient and cost‐effective solution for long‐term functional assessment and toxicity screening in OoC systems, promoting practical applications of this technology in drug development.

These two studies collectively demonstrate the technical strengths of multi‐organ chip systems through two distinct dimensions—metabolomic analysis and real‐time monitoring—thereby providing crucial methodological support for enhancing both the efficiency and success rates of drug development.

### 3.4 Application of Other Omics Techniques in Diseases

In the field of pulmonary research, scientists have successfully developed a multicellular 3D lung‐on‐a‐chip system capable of accurately simulating the dynamic pulmonary microenvironment [145]. This system utilizes computer‐aided design and 3D‐printed molds to fabricate a PDMS‐based chip platform with specialized compartments, enabling spatially precise co‐culture of small airway epithelial cells, vascular endothelial cells, stromal cells, and macrophages. The research team innovatively integrated the green fluorescent protein gene into the regulatory sequence of the pulmonary toxicity biomarker SERPINB2, establishing a fluorescent reporting platform for visual quantitative assessment of drug‐induced lung toxicity. The system demonstrates not only high sensitivity and specificity but also provides a reliable tool for early pulmonary toxicity detection. Its modular design further supports simulation of various pulmonary diseases and drug evaluation, offering robust technical support for respiratory system research.

In the field of host–microbe interaction research, the anaerobic gut‐on‐a‐chip system developed by Jalili‐Firoozinezhad's team [146] represents a significant breakthrough. This dual‐channel PDMS device, integrated with micro‐oxygen sensors and an anaerobic chamber, successfully maintained oxygen concentrations below 0.3% at the epithelial interface, achieving for the first time stable co‐culture of complex human gut microbiota with intestinal epithelial cells. The system not only preserved microbial diversity comprising over 200 operational taxonomic units but also promoted normal epithelial differentiation and barrier function, with notably enhanced barrier integrity observed under co‐culture conditions with Bacteroides fragilis. This innovative model effectively addresses the technical challenge faced by conventional in vitro systems in simultaneously supporting long‐term co‐culture of anaerobic microbial communities and host cells, providing a novel experimental platform for mechanistic studies and drug screening of microbiome‐related therapies.

## 4 Challenges and Limitations

Although the integration of OoC technology with multi‐omics approaches has demonstrated tremendous potential, it still faces dual bottlenecks at both technical and biological levels. This section systematically outlines the core challenges: Technically, it focuses on issues such as the lack of unified standards for chip design and culture conditions, insufficient sensitivity of multi‐omics detection for microscale samples, difficulties in integrating high‐dimensional heterogeneous data, and prohibitive technical costs; biologically, it analyzes key pain points including the predicament of integrating immune components and microbiomes into models, and the maintenance of long‐term stability in multi‐organ chip systems. Simultaneously, it presents targeted solutions currently available in the field, aiming to provide a clear direction for technical optimization and breakthroughs.

### 4.1 Technical Challenges

OoC technology still lacks unified standards, which constitutes the primary obstacle to its reproducibility and widespread adoption. The wide variation in chip design—including materials (e.g., PDMS may adsorb small molecules), dimensions, and architecture—has led to difficulties in directly comparing data across laboratories [149]. Disparities in cell sources (such as primary cells, cell lines, or iPSC‐derived cells) and batch‐to‐batch variability significantly impact the stability and reliability of the models [150]. Moreover, precise control and standardization of culture conditions—such as medium composition, flow rate, and shear stress—pose substantial challenges [151]. Perhaps most critically, engineering highly complex organ systems that accurately mimic inter‐organ interactions—such as “body‐on‐a‐chip” models incorporating immune cells, innervation, and microbiomes‐remains enormously difficult from both engineering and biological perspectives [152]. To address standardization challenges, the field is increasingly promoting the establishment of open‐source chip designs and standardized operating procedures (SOPs). In terms of materials, novel inert alternatives to PDMS—such as polystyrene and cyclic olefin copolymers—are being explored to minimize molecular adsorption [153]. For cell sources, efforts are underway to establish high‐quality iPSC banks and standardized differentiation protocols to improve consistency and reproducibility [154]. Regarding complex multi‐organ systems, a modular strategy is being adopted: Individual organ modules are first standardized, then interconnected via interoperable interfaces to progressively construct more sophisticated systems [155].

The miniaturized nature of OoC platforms results in extremely limited sample volumes (both in terms of cell numbers and supernatant), placing exceptionally high demands on the sensitivity of multi‐omics analyses [156]. Conventional omics technologies typically require large sample inputs, making comprehensive genomic, transcriptomic, proteomic, and metabolomic coverage of single or few cells from microliter‐to‐nanoliter‐scale chip samples a core technical bottleneck—without sacrificing information depth [157]. A direct approach to addressing this bottleneck lies in developing ultra‐high‐sensitivity detection technologies compatible with miniaturized platforms. For example, single‐cell RNA sequencing (scRNA‐seq) enables direct resolution of cellular heterogeneity within chips [158, 159]. Similarly, microfluidics‐coupled mass spectrometry and low‐input proteomic methods can reduce required sample volumes to the nanoliter scale, achieving deep coverage of trace‐secreted proteins and metabolites [160].

The field generates quintessential big data that are multi‐modal and high‐dimensional. Effectively integrating data from genomics, transcriptomics, proteomics, and metabolomics with physical parameters recorded by the chips—such as barrier function and contractile force—to extract meaningful biological insights poses a significant bioinformatics challenge [161]. This necessitates the development of novel computational algorithms and artificial intelligence (AI) models to elucidate causal networks across different molecular layers, moving beyond mere correlation to truly elevate insights from data to mechanism [162, 163]. Addressing this data complexity relies on adopting advanced computational algorithms and AI models. Approaches such as multimodal deep learning and graph neural networks (GNNs) are being employed to integrate heterogeneous data and infer causal network relationships across molecular layers, rather than simple correlations [164]. Concurrently, the development of specialized bioinformatics software pipelines and the construction of public databases aim to standardize data analysis workflows and facilitate data sharing and comparison [165].

The widespread adoption of this technology is significantly constrained by prohibitive costs. These include expenses for precision chip fabrication, advanced instrumentation for system operation (e.g., microfluidic control systems), substantial outlays for multi‐omics analyses (particularly single‐cell sequencing and high‐resolution mass spectrometry), coupled with the costs of powerful computational resources and specialized bioinformatics expertise required for subsequent data analysis [166]. These factors collectively hinder many research institutions from adopting the technology, impacting its democratization and scalable application [167]. In parallel, efforts to reduce costs and enhance accessibility are focusing on multiple fronts. These include promoting low‐cost manufacturing techniques (e.g., 3D printing), developing commercial ready‐to‐use chips to lower the entry barrier, and establishing shared core facilities, enabling broader access to advanced instrumentation and computational resources at manageable costs [15].

### 4.2 Biological Challenges

Beyond technical bottlenecks, OoC systems face profound biological challenges that directly impact their relevance in modeling human physiology and pathology.

Beyond technical hurdles, OoC systems confront significant biological challenges that directly impact their physiological relevance. Most current models primarily focus on simulating parenchymal and stromal cells of specific organs while generally lacking integrated immune components and human microbiome elements, substantially limiting their physiological fidelity [168, 169]. Immune cells play crucial roles in inflammation, tissue repair, tumor immune surveillance, and drug responses [170]. Similarly, resident microbial communities in organs like the gut, skin, and lungs critically influence both local and systemic immune‐metabolic homeostasis [171, 172, 173]. Therefore, how to stably introduce circulating or tissue‐resident immune cells into chips while maintaining their functionality, and successfully colonize specific human microbial communities in corresponding organ chips (such as gut‐on‐a‐chip), represents essential challenges that must be overcome for constructing next‐generation highly biomimetic models. Although some studies have attempted co‐culturing microbial communities in gut chips [146], maintaining long‐term microbial balance and simulating their complex interactions with host cells remains particularly challenging. Researchers are addressing these limitations through multiple strategies. One approach involves creating vascularized channels by designing vascular compartments containing human endothelial cells within chips, enabling controlled introduction of circulating immune cells (e.g., peripheral blood mononuclear cells‐PBMCs) to simulate immune cell recruitment and inflammatory responses [120]. Another strategy incorporates tissue‐resident immune cells, such as co‐culturing macrophages or regulatory T cells in gut chips to study local immune tolerance [174]. For microbiome integration, establishing anaerobic symbiotic microenvironments is crucial [175]. For instance, by designing independent, precisely controlled anaerobic microbial chambers adjacent to intestinal epithelial compartments and utilizing micropumps to regulate flow rates, researchers have successfully achieved long‐term (over 5 days) stable co‐culture of human gut microbiota and host‐microbe interaction studies [146].

To simulate the integrity of human systems, multi‐organ chip systems interconnect modules representing different organs via microfluidic channels, enabling the investigation of systemic drug distribution, metabolism, and toxicity [176, 177]. However, maintaining the long‐term stability of inter‐organ interactions within such systems poses a significant challenge. Cells from different organ types require distinct optimal culture conditions (e.g., medium composition, flow rates), making it difficult to satisfy all cellular needs simultaneously when using a shared circulating medium. This incompatibility can lead to accelerated functional decline in certain organ modules [178]. Furthermore, ensuring synchronized functional maturation and stability across various engineered tissues over weeks or even months, while accurately reproducing hormone and cytokine‐mediated inter‐organ communication, imposes stringent demands on materials, fluidic control, and life support systems. Strategies to address stability challenges focus on customized media and dynamic regulation. One approach employs a “universal medium”—a compromised formulation identified through screening and optimization that basically supports the function of all connected organs [179]. A more advanced strategy involves developing modular, interchangeable systems, allowing each organ module to be cultured under independent optimized conditions. These modules are then intermittently connected and sampled via automated liquid handling systems, thereby mitigating conflicts arising from long‐term shared circulation [155]. In addition, integrating online, non‐invasive sensors to continuously monitor metabolic parameters (e.g., glucose, lactate, and pH) in each module, coupled with dynamic feedback regulation of culture conditions, represents a critical technical direction for maintaining long‐term stability [180].

## 5 Conclusion and Prospects

OoC technology integrated with multi‐omics approaches is currently in a phase of rapid development, with the core objective of constructing more physiologically relevant, dynamically responsive, and informationally comprehensive biomimetic systems. A key future direction involves advancing “Human‐on‐a‐Chip” systems, which interconnect more than ten organ chip modules via microfluidic networks to simulate systemic compound exposure, distribution, metabolism, and tissue‐specific toxicity, thereby providing a holistic perspective for drug evaluation. However, after achieving the interconnection of 4–7 organ modules, maintaining long‐term stability in whole‐body‐level simulations remains a major challenge for the future [181, 182]. Another significant trend is the integration of in situ and real‐time monitoring technologies into the chip architecture, such as embedding miniature biosensors or microelectrodes. This enables continuous, non‐invasive monitoring of metabolites and protein biomarkers like glucose, lactate, and cytokines. Such technological progress allows dynamic capture of rapid molecular responses to drug interventions or disease progression and provides high temporal‐resolution data for constructing “digital twin” models [183]. Simultaneously, combining high‐resolution live‐cell imaging with spatial omics technologies—such as spatial transcriptomics and CODEX imaging—enables precise mapping of gene expression and protein localization to specific functional regions of the chip (e.g., vascular lumen, epithelial layer). This preserves the native spatial architecture and facilitates in‐depth analysis of biological processes such as tumor heterogeneity and immune cell infiltration [184, 185, 186]. At the technical level, imaging methods based on antibody‐fluorescence labeling and high‐resolution microscopy (e.g., CODEX, MIBI) allow simultaneous in situ visualization of dozens of proteins [187, 188]. Meanwhile, spatial transcriptomics technologies utilizing single‐molecule fluorescence in situ hybridization (smFISH) imaging (e.g., MERFISH, Seq‐Scope) can resolve the spatial distribution of hundreds to thousands of mRNA species within tissues at subcellular resolution [189, 190]. Furthermore, multimodal data integration, which combines proteomic, transcriptomic, conventional H&E staining, or live‐cell imaging data to construct high‐dimensional spatial maps, represents the latest research direction. This approach reveals cellular interaction networks and microenvironmental dynamics in unprecedented detail [191, 192].

As OoCs and multi‐omics data continue to grow in both scale and complexity, AI will become the core driver for extracting their deeper value and enabling predictive capabilities. Its applications will extend from data integration to constructing predictive virtual models. Faced with the massive, high‐dimensional, and heterogeneous data generated by high‐throughput experiments—such as transcriptomics, proteomics, metabolomics, and physical readouts from chips—traditional bioinformatics methods are increasingly inadequate [193]. Machine learning (ML) and deep learning (DL) algorithms can efficiently extract key features and identify underlying patterns from these complex datasets [194]. For instance, GNNs can model different molecular entities and their interactions as complex networks, thereby uncovering cross‐omics regulatory pathways [195]. Unsupervised learning methods, such as variational autoencoders (VAE), can identify novel cell states or biomarkers within high‐dimensional data without prior knowledge [196]. The ultimate goal is to integrate these multimodal datasets to construct “digital twin” models of organs or diseases—virtual entities capable of dynamically simulating and predicting biological system responses to perturbations, such as drug treatments [197]. A more advanced application of AI involves integrating OoCs experimental data with physical laws and mechanistic mathematical models to build virtual organ or virtual patient models [198]. Such models not only describe “what” happens but also explain “why”. For example, combining PK models with experimental data from multiple organ chips and using AI to calibrate parameters can virtually predict the concentration‐time profiles and potential toxicity of new compounds across different organs, significantly reducing the number of experimental screening rounds [199]. In personalized medicine, leveraging patient‐specific cell‐derived OoCs data to individually calibrate such models enables the creation of “virtual patients” for predicting individual responses to different treatment regimens, providing critical decision support for achieving true precision medicine [200].

In the field of regenerative medicine, organ‐on‐a‐chip technology provides a robust screening and validated platform for optimizing tissue engineering strategies [201, 202]. This technology can precisely simulate in vivo biomechanical and biochemical microenvironments to test the effects of novel biomaterials, stem cell derivatives, or growth factors on tissue formation and function [203, 204]. For instance, it can be applied to evaluate how scaffold materials for cartilage or vascular regeneration support directed cell differentiation and functional tissue assembly, thereby accelerating the development of safer and more effective regenerative therapies [205]. Meanwhile, in regulatory science, a clear direction involves promoting OoC systems combined with multi‐omics data as alternatives or supplements to animal testing, ultimately seeking acceptance from regulatory agencies such as the U.S. FDA and the European EMA. This shift is supported by policy initiatives, such as the U.S. FDA Modernization Act 2.0, which encourages the adoption of emerging technologies to replace animal testing [206]. By generating standardized, reproducible, and clinically relevant multi‐omics data, organ‐on‐a‐chip platforms are expected to provide more human‐relevant evidence for evaluating drug safety and efficacy, thereby reshaping the paradigms of toxicology testing and drug regulatory review [207].

In summary, the convergence of OoCs and multi‐omics technologies represents a revolutionary shift in our approach to understanding human biology and developing therapeutics. As technical and analytical challenges are addressed, this platform is expected to fundamentally reshape the drug development landscape over the next decade. It will establish a new, more efficient R&D paradigm—“OoCs screening → mechanistic validation → precision clinical trials”—and powerfully advance the transition from population‐based medicine to individualized precision healthcare.

## Author Contributions

X. C. and C. X. prepared the figures and drafted the manuscript. C. L., S. D., and J. C. edited and revised the manuscript. H. Y., S. W., and P. C. approved the final version of manuscript. All authors have read and approved the final manuscript.

## Ethics Statement

The authors have nothing to report.

## Conflicts of Interest

The authors declare no conflicts of interest.

## Data Availability

The data that support the findings of this study are available from the corresponding author upon reasonable request.
